# Draft Genome Assembly of a Potentially Zoonotic Cryptosporidium parvum Isolate, UKP1

**DOI:** 10.1128/MRA.01291-18

**Published:** 2018-11-15

**Authors:** John H. E. Nash, James Robertson, Kristin Elwin, Rachel A. Chalmers, Andrew M. Kropinski, Rebecca A. Guy

**Affiliations:** a National Microbiology Laboratory, Public Health Agency of Canada, Guelph, Ontario, Canada; b Singleton Hospital, *Cryptosporidium* Reference Unit, Public Health Wales Microbiology, Swansea, United Kingdom; c Department of Pathobiology, Ontario Veterinary College, University of Guelph, Guelph, Ontario, Canada; University of Maryland School of Medicine

## Abstract

Cryptosporidium parvum is a zoonotic protozoan parasite that causes food and waterborne gastrointestinal disease and whose major animal reservoirs are cattle and small ruminants. We report here on a draft whole-genome sequence of a zoonotic isolate of C. parvum isolated from a person with cryptosporidiosis.

## ANNOUNCEMENT

Cryptosporidium parvum is one of the two major species of the protozoan parasite Cryptosporidium that infect humans and cause gastrointestinal disease (1). C. parvum is considered zoonotic and has a wide host range, in which cattle and small ruminants are the predominant reservoir hosts (2). However, some subtypes of C. parvum, such as the IIc gp60 subgroup, are considered to be human adapted (2), because they are mainly reported in humans, with only a few reports in European hedgehogs (3). Whole-genome sequencing (WGS) provides a means for comparing isolates to identify markers important in distinguishing routes of transmission and potential virulence traits for better epidemiological analysis and risk assessment. The objective of this work was to sequence a zoonotic isolate of C. parvum.

We sequenced a human isolate of C. parvum, UKP1, isolated at the Public Health England and Public Health Wales Cryptosporidium Reference Unit and identified as gp60 type IIaA17G1R1 (GenBank accession no. JX971701). This subtype has a global distribution and has been observed in humans, cattle, pigs, and sheep (4, 5). Zoonotic linkages with cases in England and Wales have been demonstrated with IIaA17G1R1 (4). Oocysts were semipurified from stool using a salt flotation and hypochlorite treatment. DNA was extracted with the QIAamp DNA extraction kit (Qiagen, Hilden, Germany) and whole-genome amplified with the REPLI-g kit (Qiagen) before 454 GS FLX Titanium and Illumina HiSeq 2500 sequencing. A total of 472.6 Mbp, representing 1.6 million reads, were obtained from the 454 GS FLX Titanium sequencing, and 12.8 Gbp, representing 26 million reads, were produced on an Illumina MiSeq instrument. The quality of the reads was examined using FastQC (6). Illumina and 454 reads were mapped against a reference C. parvum isolate (GenBank accession no. NZ_AAEE00000000) using Bowtie 2 v. 2.3.3.1 (7), and the mapped reads were then *de novo* assembled using SPAdes v. 3.11.0 (8). The initial assembly was used in a second round of assembly using the --trusted-contigs flag with SPAdes. Iterative polishing of the assembly was done by mapping the reads back to the assembly with Bowtie 2 and correcting them with Pilon 1.22 (9). The 8 C. parvum chromosomes assembled into 14 contigs with a total genome size of 8,881,956 bp, a G+C content of 30.20%, an *N*_50_ value of 1,092,230 bp, and a largest contig length of 1,333,759 bp.

### Data availability.

This whole-genome shotgun project has been deposited at DDBJ/EMBL/GenBank under the accession no. PYCJ00000000, and raw sequence reads are available under the BioProject no. PRJNA439211.
